# Age 20 Cognitive Ability Moderates the Long-Term Influence of Lifestyle Behaviors on Brain Aging in Late Midlife

**DOI:** 10.1093/geroni/igaa057.384

**Published:** 2020-12-16

**Authors:** Carol Franz, Teresa Warren, William Kremen

**Affiliations:** 1 University of California San Diego, La Jolla, California, United States; 2 San Diego State University, La Jolla, California, United States; 3 University of California San Diego, San Diego, California, United States

## Abstract

We examined whether the longitudinal association between lifestyle behaviors and brain age is moderated by early general cognitive ability (GCA). The sample comprises 356 participants from the Vietnam Era Twin Study of Aging (VETSA). At mean age 40 (SD 2.7; range 34-44) a positive lifestyle index was created comprising three self-reported behaviors: not smoking, zero to moderate alcohol consumption, and high social engagement. GCA at mean age 20 was assessed with the Armed Forces Qualification Test. At mean age 68 (SD 2.6; range 61-72), participants underwent structural magnetic resonance imaging which was used to create predicted brain age difference (PBAD) scores. Multivariate models included GCA, lifestyle and their interaction as IVs, adjusted for age, ethnicity, APOE genotype, height, and family membership. Age 20 GCA and age 40 lifestyle significantly predicted age 68 PBAD [F=5.83; p=.02 and F=15.14; p<.001, respectively]. Both positive behaviors and higher age 20 GCA were associated with less brain aging. The GCA-lifestyle interaction was also significant. Those with both lower age 20 GCA and fewer positive behaviors had older brains relative to chronological age [F=5.00; p=. 03]. When GCA was high, however, participants had younger brains, regardless of lifestyle behaviors, suggesting a protective effect of early high GCA or cognitive reserve on later brain health. However, for those with lower cognitive reserve, positive lifestyle behaviors appeared to be protective against brain aging nearly three decades later. Results highlight the important role of cognitive reserve and lifestyle factors for later life brain health.

